# Psychological Considerations in the Dietary Management of Patients With DGBI

**DOI:** 10.14309/ajg.0000000000001766

**Published:** 2022-04-08

**Authors:** Helen Burton Murray, Bethany Doerfler, Kimberly N. Harer, Laurie Keefer

**Affiliations:** 1 Harvard School of Medicine, Boston, Massachusetts, USA;; 2 Center for Neurointestinal Health, Massachusetts General Hospital, Boston, Massachusetts, USA;; 3 Feinberg School of Medicine, Northwestern University Medical Center, Chicago, Illinois, USA;; 4 University of Michigan, Ann Arbor, Michigan, USA;; 5 Icahn School of Medicine at Mount Sinai, New York, New York, USA.

## Abstract

In this article, an expert team of 2 gastro-psychologists, a dietician, and an academic gastroenterologist provides insights into the psychological and social implications of evidence-based and “popular” dietary interventions in disorders of gut-brain interaction (DGBI). We focus on practical approaches for evaluating a patient's appropriateness for a dietary intervention, considering the nutritional, psychological, behavioral, and social context in which a patient may find themselves managing their DGBI with dietary intervention. We also discuss how to identify risk factors for and symptoms of avoidant/restrictive food intake disorder, a growing concern in the DGBI population.

## INTRODUCTION

Dietary interventions for the management of symptoms associated with disorders of gut-brain interaction (DGBI) are increasingly prescribed as part of integrated care (1), with varying levels of evidence or scientific premise (see review in this edition) (2). For example, gluten-free and lactose-free diets are commonly recommended for patients with irritable bowel syndrome (IBS) (2), despite limited understanding of the role these foods play in the pathogenesis or maintenance of DGBI symptoms, especially over the long term. The low fermentable oligosaccharides, disaccharides, monosaccharides and polyols (FODMAP) diet may have a stronger scientific premise in DGBI (specifically for IBS) (3–5) but without properly designed meal planning, and a plan for food reintroduction could be harmful over the long term (6,7). Several “popular diets” have also been adopted by patients with DGBI that involve significant food restriction with little or no scientific justification and could worsen symptoms (e.g., excess fructose consumption in the Whole 30 diet) or other health outcomes (e.g., higher saturated fat intake in “the Plant Paradox” diet) (8,9).

Patient-provider collaboration around the choice of dietary intervention is critical to the proper uptake and safety of any dietary intervention—these may include a patient's expectations for risks and benefits because they relate to symptom improvement, quality of life and emotional well-being, as well as agreement on the anticipated duration of the diet and any follow-up requirements. Whenever possible, a registered dietitian (RD) should be included as part of the patient's care team. Health psychologists may also be helpful in assisting the care team in the choice of dietary intervention, improving adherence and supporting lifestyle change, as well as identifying disordered eating behaviors, eating disorders, or other contraindications to restrictive dietary interventions.

**Clinical pearl #1: registered dietitians—experts in nutrition assessment and execution of medically indicated nutrition care plans**

Registered dietitian nutritionists (RDNs) are nutrition content experts who are nationally and locally licensed to deliver medical nutrition therapy to individuals with medical conditions including IBS and DGBI. By way of training, RDNs complete a minimum of a bachelor's degree at an accredited university or college and course work approved by the Accreditation Council for Education in Nutrition and Dietetics of the Academy of Nutrition and Dietetics (AND), and beginning in 2024, RDNs will be required to complete a graduate degree. Additional advanced areas of practice and certification for RDNs are unique to this field. Currently, in many states, RDNs are the only nutrition professionals who are licensed to provide nutrition care plans. In contrast to RDNs, a nutrition professional who is a nutritionist may have limited experience with DGBI and nutrition assessment. Recent partnerships with American Gastroenterological Association (AGA) and the AND deliver nutritionally evidence-based content to gastrointestinal (GI) clinicians across GI disease spectrum. Several certification projects exist for RDN to be specifically trained in medical diets such as the low FODMAP diet (e.g., Monash University FODMAP and IBS training).

**Figure 1.**
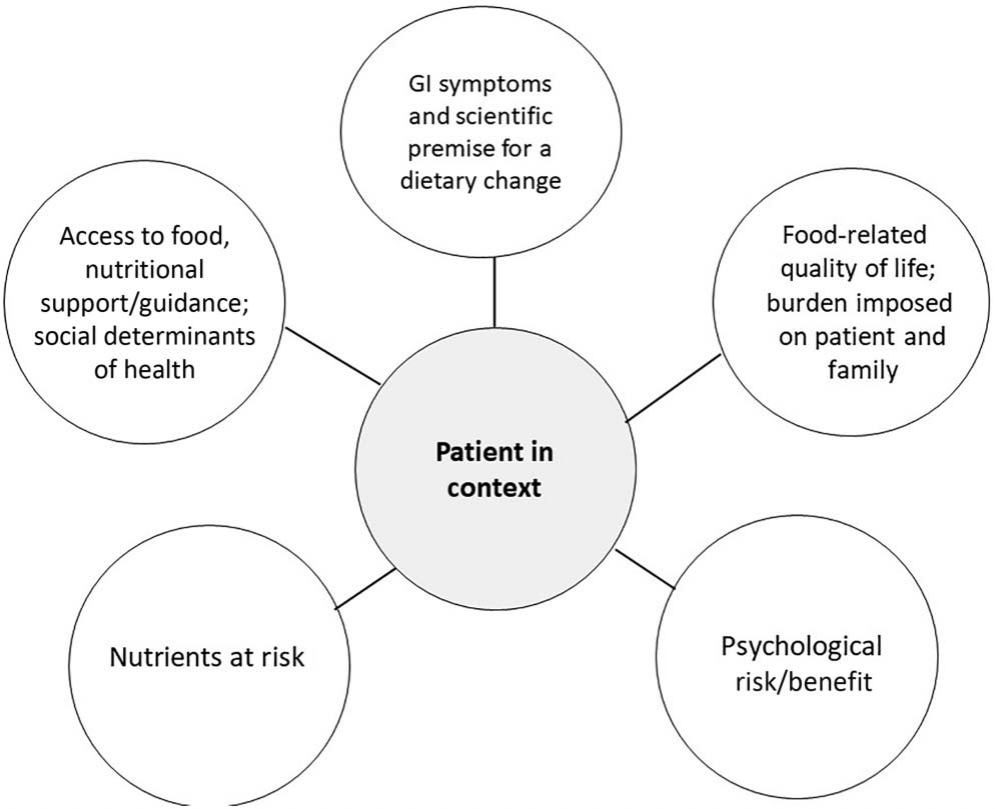
Shared decision-making around diet requires an understanding of the patient in the biopsychosocial context. The nutritional needs of a patient with DGBI, along with the specific symptoms being targeted, must always be considered when recommending a dietary intervention for a patient with DGBI. However, the psychological and quality-of-life risks and benefits as well as social determinants of health including access to food and nutritional support must not be separated from the shared decision-making process. DGBI, degrees of expert guidance.

In this article, we provide insights into the psychological and social implications of evidence-based and popular dietary interventions in DGBI. We focus on practical approaches for evaluating a patient's appropriateness for a dietary intervention, considering the nutritional, psychological, behavioral, and social context in which a patient may find themselves managing their DGBI with dietary intervention (Figure 1). Below, we delineate practical approaches for (i) managing patient expectations around the role of food allergy and motility testing, (ii) evaluating the scientific premise for food elimination or restriction with the patient's psychological and nutritional risks and benefits in mind, (iii) recognizing risk for disordered eating, and (iv) developing a pathway for low-resource patients to access safe dietary interventions when appropriate.

### Managing patient expectations around the role of food allergy and motility testing to inform dietary intervention choice

The psychological and nutritional impact of GI testing is an important consideration when evaluating patients with GI symptoms. This is particularly relevant when considering food allergy testing. Previous studies have shown that a diagnosis of a food allergy is associated with increased food anxiety, social isolation, and decreased quality of life (10–13). Thus, it is important to focus on evidence-based allergy testing methods and educate patients about the pitfalls of other testing methods.

For example, oral food challenge, skin prick testing, and serum Immunoglobulin E (IgE) testing are the gold standard methods of food allergy testing when food allergy symptoms are present. Serum Immunoglobulin G (IgG) testing and commercially available food sensitivity panels are increasing in popularity but should be approached with extreme caution because of the lack of clinical relevance and the negative impact a positive result may have on dietary restriction and quality of life. Serum IgG testing and antigen leukocyte antibody test are not recommended to diagnose food allergies, hypersensitivities, or intolerances because of low test specificity and poor reproducibility (14). Although a positive serum IgG or antigen leukocyte antibody test does not indicate a food allergy or sensitivity, patients often interpret the test result as an allergy, which can subsequently result in labeling those foods as unsafe and harmful and drive the implementation of unnecessary food restrictions. Thus, routine food allergy testing in the absence of true allergy symptoms is not recommended. Evaluation of food sensitivities or intolerances by using any method is not recommended for managing patients' DGBI symptoms; however, guided dietary therapy to identify food triggers is recommended. We offer some example terminology to aid providers in discussing food allergy tests with patients in Table 1.

**Table 1.**
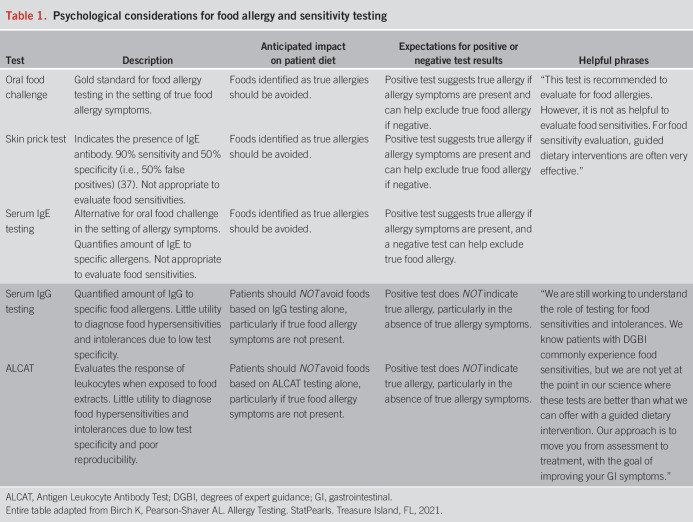
Psychological considerations for food allergy and sensitivity testing

GI-associated tests to evaluate DGBI symptoms as they relate to eating behavior can also have negative psychological and dietary impact, regardless if the test is normal or abnormal. Abnormal testing can result in rash dietary changes (e.g., gastric emptying study resulting in recommendation of implementing a restrictive gastroparesis diet). Alternatively, the psychological impact of negative testing should also be appreciated. Negative testing can result in disappointment and frustration because of a lack of identified etiology for the patient's suffering and symptoms. The frustrations of multiple normal test results are also amplified in the DGBI population secondary to the paucity of diagnostic testing available for motility and functional disorders because many DGBI diagnoses occur *after* negative tests evaluating for other diagnoses. It is important for providers to set expectations regarding what a positive or negative test result will mean and reassure patients that their symptoms will continue to be treated, regardless of test results.

### Evaluating the scientific premise for food elimination or restriction with the patient's psychological and nutritional risks and benefits in mind

It is crucial to weigh the benefits of dietary interventions in the context of (i) nutrients and calories at risk, (ii) the time frame or duration of the dietary intervention, and (iii) the behavioral risk factors, social implications (e.g., access, cost, and cultural practices), and the impact of the diet on quality of life. These considerations to patients who are already following a diet that you would like them to liberate, or for patients who are asking about a diet they could follow for immediate symptom relief. Table 2 defines and highlights possible risks associated with popular diets that may be commonly self-initiated by patients (Table 2) and evidence-based diets that may be either self-initiated by patients or medically prescribed (Table 3) to treat DGBI.

**Table 2.**
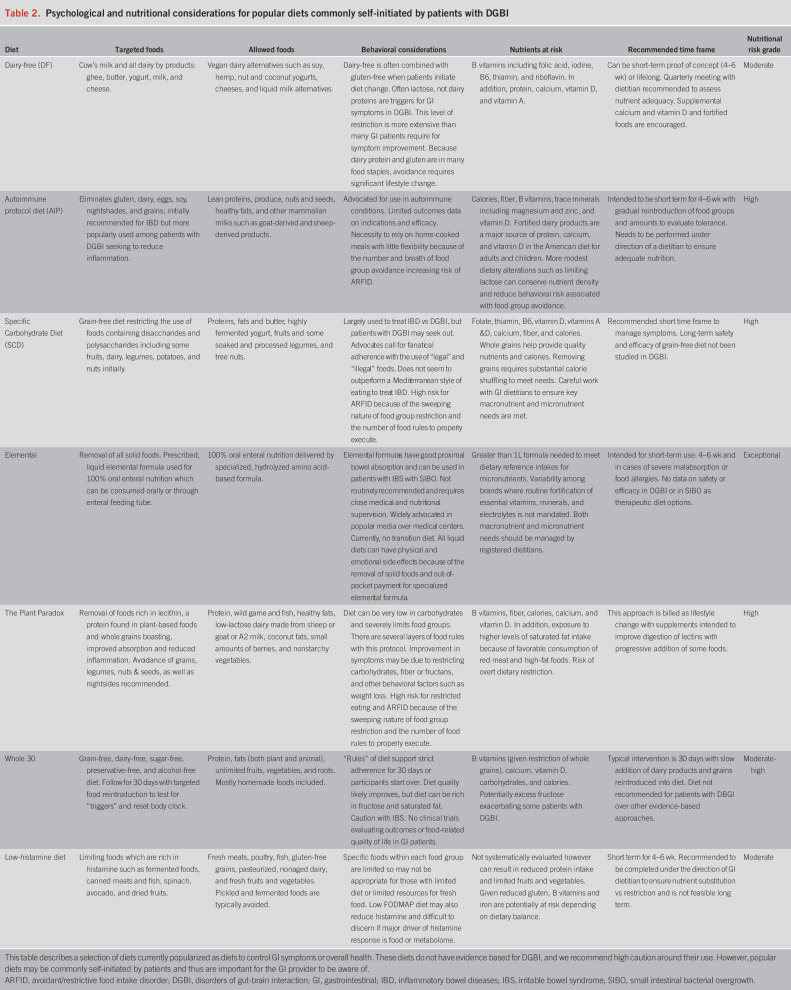
Psychological and nutritional considerations for popular diets commonly self-initiated by patients with DGBI

**Table 3.**
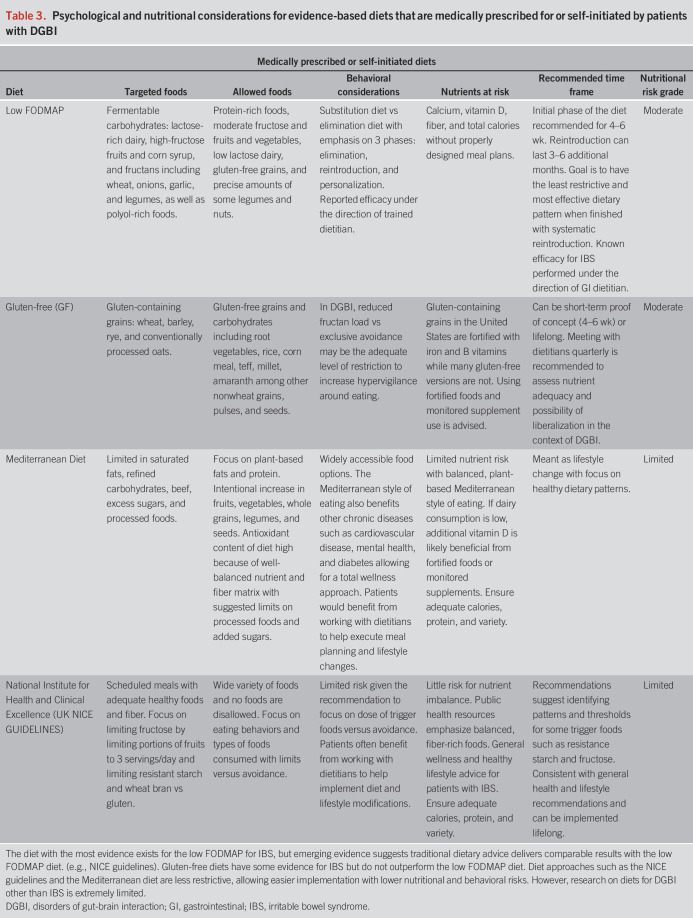
Psychological and nutritional considerations for evidence-based diets that are medically prescribed for or self-initiated by patients with DGBI

#### Nutrients and calories at risk.

Dietary restriction is usually associated with nutrient deficiencies that should be monitored and potentially supplemented—for example, several nutrients are at risk in a gluten-free diet, including folic acid, B6, thiamin, riboflavin, niacin, iron, and dietary fiber—these may need to be assessed quarterly and supplemented as appropriate if the patient is on the long-term diet. Similarly, calories are often decreased in restrictive diets, increasing risk for malnutrition and, in some cases, an eating disorder (15,16). Weight loss is not an intended outcome with diets for DGBI and should also be monitored (Table 4). The low-histamine diet has not been evaluated in DGBI but limits protein intake as well as fruits and vegetables, posing other health risks and malnutrition. Diets where nutrients and calories are potentially lower risk may include the Mediterranean diet, which is also widely accessible and has long-term health benefits outside of GI (17).

**Table 4.**
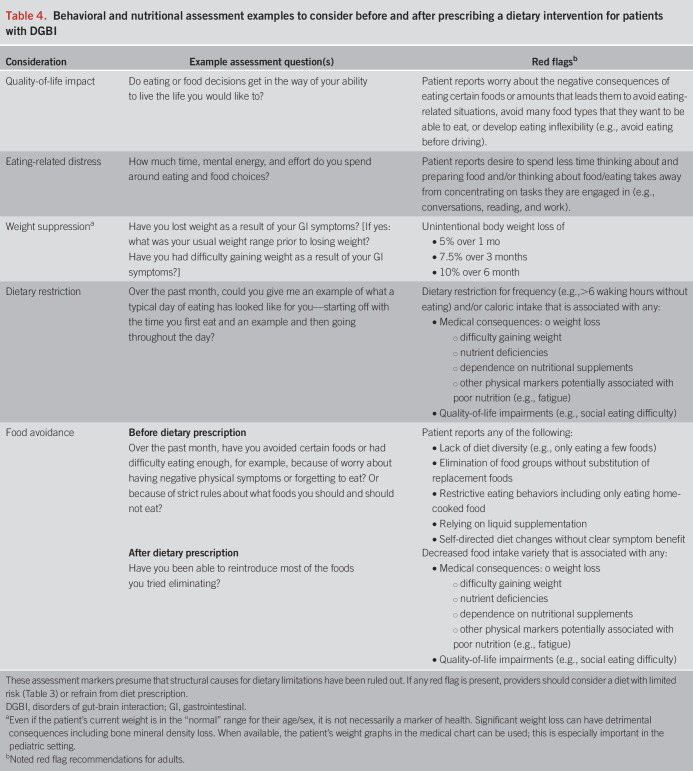
Behavioral and nutritional assessment examples to consider before and after prescribing a dietary intervention for patients with DGBI

#### Time frame.

Several diets are empirically supported for DGBIs (primarily the low FODMAP diet for IBS) but are not intended to be followed indefinitely. It is important to communicate with your patient about the time frame in which you are expecting them to begin reintroducing foods and liberate their diet. For example, a gluten-free diet may be tried empirically for 4–6 weeks with a motivated, low-risk patient with the expectation that if symptoms are not significantly improved, the patient would reintroduce gluten as part of a balanced diet. Another example is the low FODMAP diet, in which the initial restrictive phase is intended for only 4–6 weeks, with a reintroduction period also short, between 3 and 6 months. Again, the patient and provider must agree that ultimately, the shared goal is the least restrictive diet that manages their symptoms.

**Clinical pearl #2: managing the patient interested in or on a fad diet**

Below are some red flags for fad (or popular) diets that include strict and dogmatic approaches to limiting whole food groups to improve symptoms. See Table 4 for additional guidance.

1. Popular diets (Table 2) can overpromise physiologic benefits and may involve sophisticated rituals related to eating and cooking. Often there is theoretical evidence without studies showing safety and efficacy.
2. Patients with DGBI are often interested in integrating both conventional medical care and complementary approaches such as herbal therapies, dietary modifications, and other supplements.
3. Social media personalities who experienced “amazing results” can be powerful influencers for patients wanting relief. Undoubtedly, popular diets can drive both physical and psychological harm to patients because of the perception that stricter adherence equals better results. This subsequently sets the stage for problematic cognitive/emotional (e.g., guilt or fear around eating) and behavioral (e.g., binge eating or social eating avoidance) outcomes, which negatively affect functioning and/or nutritional status.
4. Patients will often seek approval and/or guidance from medical providers on their approach. Clinicians can steer patients away from the more dangerous elements of popular diets by validating the role of food intolerances in DGBI and by reassuring that less restrictive, evidence-based approaches have been well studied and do produce favorable results in many.
5. Involving a dietitian allows the focus to be on what patients *can* eat versus what they “cannot.” The nutritional goal is always to provide the least restrictive and most varied diet modification which minimizes symptoms and optimizes diet quality.
6. Dismissive counseling or shaming patients for exploring alternatives jeopardizes dynamic communication between patient and provider.

#### Behavioral and psychosocial risk.

One of the most significant behavioral considerations in dietary intervention is the impact of food selection and diet on quality of life. Poor food-related quality of life in individuals with IBS has been associated with higher levels of food avoidance (including the use of elimination diets) (18) and diminished nutrient quality (19,20). Furthermore, based on data in patients with celiac disease, a gluten-free diet may put some patients at risk for greater anxiety and somatization (21).

It is important to consider a patient's goals and lifestyle to determine whether the impact on emotional and psychological well-being is justified by the likely improvement in symptoms. For example, some common diets, such as the specific carbohydrate diet, impose significant patient emotional and financial burden and call for fanatical adherence, using terms such as “legal and illegal” foods—all this despite not outperforming more liberal diets such as the Mediterranean diet in disorders such as Crohn's disease (22). Balanced diet recommendations (e.g., Mediterranean diet and NICE guidelines) have actually shown similar outcomes to the low FODMAP diet and have higher patient acceptability (23–25). More worrisome is a patient who already reports worry about the negative consequences of eating certain foods or amounts, avoids eating-related situations, avoids foods that they want to be able to eat, or find it difficult to be flexible with eating (e.g., avoid eating before driving) may be particularly negatively affected by a restricted diet, or worse, at risk for developing an eating disorder.

### Recognizing risk for disordered eating: behavioral and nutritional assessment before and after prescribing a dietary intervention

**Figure 2.**
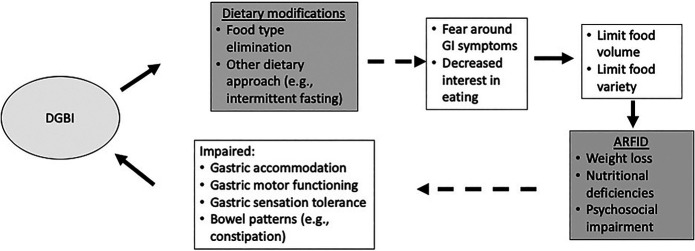
Interaction between degrees of expert guidance, restrictive diets, and avoidant/restrictive food intake disorder.

There is increasing recognition of the importance of screening for and preventing the development of maladaptive dietary restriction in patients with DGBI. In particular, a subset of patients may have dietary restriction (reduced volume, frequency, and/or variety) that crosses the eating disorder threshold as avoidant/restrictive food intake disorder (ARFID) (26,27) (Figure 2). Diet approaches for DGBI may in fact be a risk factor for ARFID, with one study showing that patients with DGBI with a history of using a diet were more than 3 times as likely to have ARFID symptoms (16).

Dietary restriction in ARFID is motivated by 1 or more reasons that is not related to body image—fear of aversive consequences (e.g., abdominal pain and diarrhea), lack of interest in eating/low appetite, and/or sensory sensitivity (e.g., taste, texture, and smell) (28). To be diagnosed with ARFID, patients must have psychosocial (e.g., difficulty with social eating) and/or medical (e.g., weight loss and nutrient deficiency) consequences of dietary restriction (26,29). Symptoms of ARFID have been reported in 13%–40% of patients with disorders of gut-brain interaction (19,27), with rates as high as 48% in IBS specifically (27). The most common motivation for ARFID restriction has been a fear of GI symptoms and occurs in individuals of all ages (30).

To identify the presence of problematic dietary restriction, we recommend providers to screen before and after dietary prescription for psychosocial and medical impacts of dietary restriction (Table 4). There are emerging self-report survey screening options for ARFID (31), but these have not yet been validated to detect ARFID in DGBI. There are no validated methods for ARFID prevention in patients with DGBI. However, before dietary prescription, providers can talk with their patients about the rates of ARFID in DGBI, how dietary restrictions are temporary (when applicable), and that the end goal is for the patient to have a nutritionally balanced diet and a flexible relationship with food.

Other eating disorders are also relevant when selecting a dietary approach for DGBI. Some patients may have a history of an eating disorder and are recovered—strict elimination diets are typically contraindicated as a risk for relapse (9). Other patients may have current cognitive (e.g., significant body image disturbance) and/or behavioral (e.g., binge eating, self-induced vomiting, and excessive exercise) manifestations of eating disorders beyond dietary restriction—elimination diets are also typically contraindicated in these cases (9). Importantly, eating disorders affect individuals of all demographics and weight status (not just those with a low weight) (32). Because the psychological effects of some dietary prescriptions for IBS can detrimentally perpetuate an eating disorder, we recommend that screening for current eating disorder symptoms should be considered for all patients with DGBI. Notably, the presence of current eating disorder symptoms does not preclude the use of dietary interventions for DGBI symptoms—modified dietary prescriptions (e.g., FODMAP “light”) can be made—and the inclusion of a multidisciplinary team is crucial (e.g., dietician monitoring + psychologist providing evidence-based eating disorder treatment). More information on assessment and treatment guidelines for eating disorders can be found in the Academy for Eating Disorders Medical Care Standards (33). A recommended short screening option is the SCOFF (34), which can be administered through clinician questioning or as a survey.

For comprehensive guidelines on identifying and managing ARFID and other eating disorders, see the work of Lemly et al. (35) and the Academy for Eating Disorders guidelines (36).

### Developing a pathway for low-resource patients to access safe dietary interventions when appropriate

Although the importance of a RD in the oversight of dietary interventions, particularly those who involve dietary restriction, cannot be overstated, we recognize that access to such services is often limited. Many patients also choose to follow dietary interventions on their own, with little guidance from professionals. Before recommending more sophisticated forms of nutritional therapy, clinicians need to consider the food environment including access to food and specialty foods as well as willingness and ability to cook. Religious and personal food practices such as vegetarianism may affect the level of dietary advice recommended. Adequate nutrition coverage for medical nutrition therapy in DGBI may influence whether patients ultimately work with a dietitian. With a recent partnership between the American Gastroenterogical Association (AGA) and the AND practice group, dietitians in GI disorders aim to provide GI disease-specific information for GI clinicians and a network of GI-trained RD for clinical and research needs. In addition, GI providers looking to integrate a RD with limited GI experience can sponsor specialized training through one of these resources.

## CONCLUSION

In this brief report, we described some of the psychological considerations influencing the choice of dietary interventions in the management of DGBI, emphasizing the importance of the gastroenterology provider in helping patients make informed decisions that consider not only nutritional and behavioral risk but also quality of life.

## CONFLICTS OF INTEREST

**Guarantor of article:** Laurie Keefer, PhD.

**Specific author contributions:** H.B.M., B.D., K.H., and L.K. all contributed to the choice of content, structure, and overall development of the manuscript draft and reviewed the final version of the document.

**Potential competing interests:** H.B.M.: receives royalties from Oxford University Press for her forthcoming book on rumination syndrome. B.D.: is on the scientific advisory board of Trellus Health and is a consultant to Reckitt Health. K.H.: no relevant disclosures or COI. L.K.: is a consultant to Abbvie, Eli Lilly, Reckitt Health, and Trellus Health. She is an equity owner Trellus Health and on the Board of Directors for the Rome Foundation. L.K. receives royalties from Routledge for her edited book *Handbook of Psychogastroenterology*.

**Financial support:** This work was supported in part by the National Institute of Diabetes and Digestive and Kidney Diseases—K23 DK131334 (H.B.M.).

**Study Highlights**

**WHAT IS KNOWN**

✓ Dietary interventions are commonly used in the management of disorders of gut-brain interaction with varying degrees of expert guidance (DGBI).
✓ Maladaptive eating behaviors and eating disorders, including avoidant/restrictive food intake disorder, are common among patients with DGBI.

**WHAT IS NEW HERE**

✓ Dietary interventions and food allergy testing should be considered in the context of psychological risk factors.
✓ Practical recommendations for the gastrointestinal provider around identifying risk and managing expectations for patients with DGBI on or interested in dietary intervention.
